# Think out of the box: revisiting sham acupuncture treatment

**DOI:** 10.3389/fneur.2024.1479239

**Published:** 2024-11-13

**Authors:** Yiu Ming Wong

**Affiliations:** Koumeido Shinkyuin Clinic, Taipei, Taiwan

**Keywords:** needle, acupoint, acupuncture, placebo, sham

## Introduction

A thousand-year-old oriental healing system, acupuncture has been described as an energy-based model of therapy in which a vital energy named “ki” travels through the body along internal channels called meridians. Fourteen meridians can be outlined on the skin surface, and 361 acupuncture points are within and run longitudinally up and down the human body. From the perspective of Asian traditional medicine, the ki obstructions in the meridians cause diseases in the body, and acupuncturists, by precisely needling the correct acupoints, can enhance the flow of ki and restore physical wellbeing (1, 2) even though the existence of the acupoints and meridians is debatable and they are not visible anatomically (3, 4). It could be reasonable to illustrate the acupuncture concept by analogy with phlebotomy or intravenous infusion, as both venous blood sampling and venous infusion can only be performed successfully when a syringe or catheter is placed properly within a targeted vein. Similarly, missing a vein or an acupoint would cause an expected effect to be lost.

## Real vs. placebo acupuncture

It has been reported that acupoints are spherical in shape, with a diameter of a few millimeters, and located in the intramuscular or periosteal layers (5, 6). A systematic review of pooled data from eight meta-analyses found that real acupuncture (overwhelmingly intramuscular needling into acupoint areas) is significantly superior to placebo acupuncture (epidermal needling into non-acupoint skin) for treating musculoskeletal pain (7). However, there were two differences between the real and the placebo acupuncture: deep (5–20 mm) vs. superficial (1–2 mm) needle insertions and classic acupoint placement vs. non-acupoint skin (8). Thus, the review above may be interpreted as proof of the advantages of deep needling for patients with musculoskeletal pain rather than the requirement that both deep needling and acupoints be precisely targeted in order to produce a therapeutic effect, because the placebo acupuncture was limited to epidermal penetration. Stated differently, is it possible that the acupoint specificity is not as essential as traditionally assumed? Without a well-designed placebo acupuncture, no study will be able to show any advantage for real acupuncture, or the study might show an advantage that may have been accidentally generated by an imperfectly designed sham procedure.

## Non-pharmacological intervention

**Figure 1:**
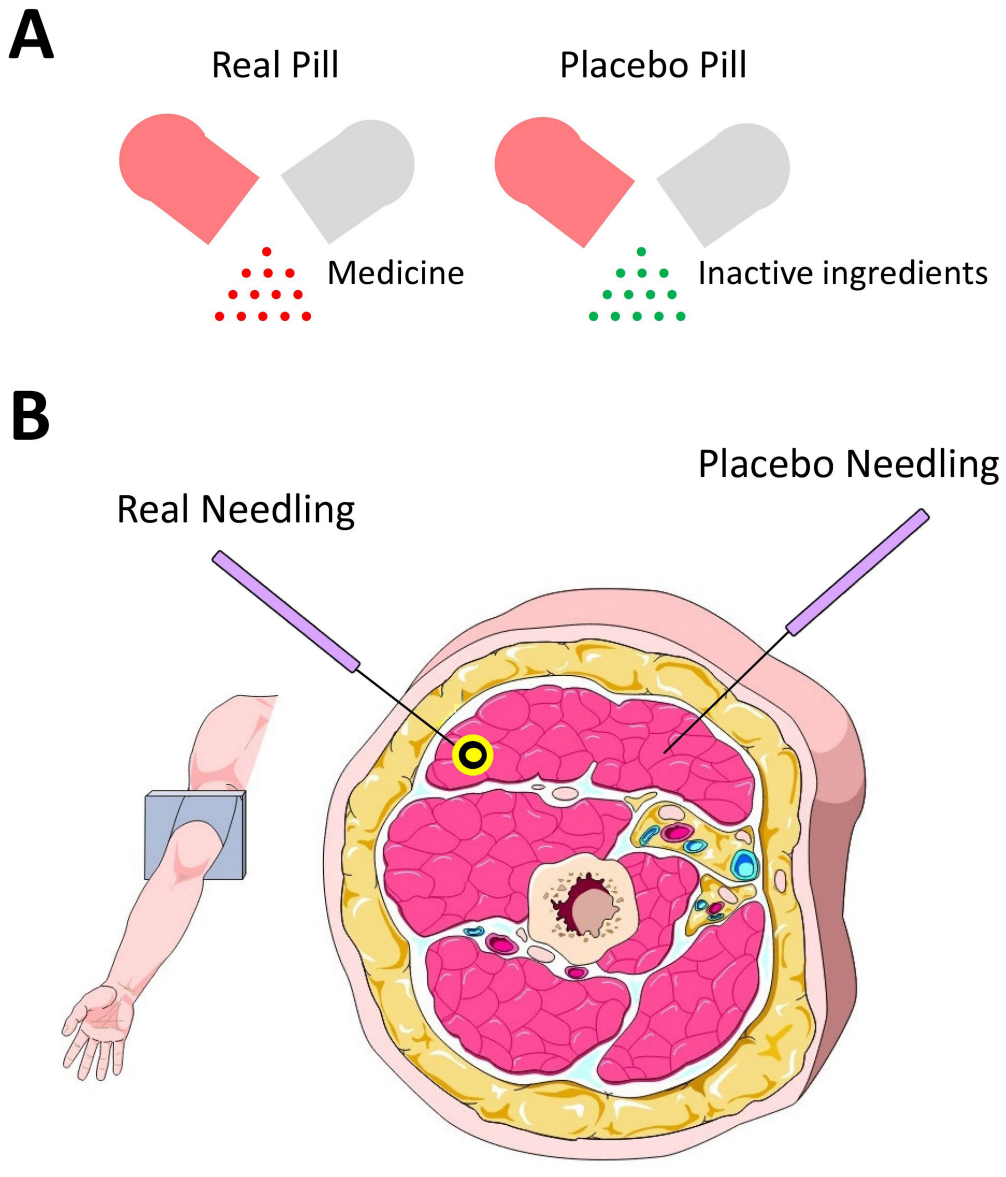
**(A)** Heterogeneity between real and placebo pills is the pill contents being active or not. **(B)** Heterogeneity between real and placebo acupuncture is the needle placements and proposed acupoint (yellow circle) being penetrated or not.

In order to confirm the efficacy of an oral medicine, researchers need to show that real medicine outperforms the placebo medicine for patients who are convinced that the placebo is the real medicine. When swallowed, the contents of the real and placebo medicines dissolve in the stomach and are absorbed into the bloodstream; thus, it can be named “substance-based intervention” (Figure 1A). Acupuncture treatment, on the other hand, is a “procedure-based intervention” which always consists of three components: (1) non-specific effects of practitioner-patient contact, e.g., skin preparation; (2) “tactile stimulation” from mechanical needling into soft tissues; and (3) “acupoint-aimed approach” as needles are inserted into the proposed acupoints. These components together can initiate complex interactions that contribute to treatment effectiveness (9). Instead, the “acupoint-aimed approach” of the aforementioned three components should be removed during the placebo acupuncture while the placebo and real needling depth should be the same (Figure 1B).

In sum of the above, the difference between real and placebo treatments can be illustrated by an equation below.

Clinical trial for efficacy of oral medicine: Capsulated medicine – Placebo capsule = Medicine.

Clinical trial for efficacy of acupuncture: Real acupuncture – Placebo acupuncture = Acupoint.

## Discussion

A critical element to judging whether or not a placebo acupuncture treatment is valid is that maximal similarity between the real and placebo treatments should be pursued (10, 11). If at all possible, the placebo acupuncture should be comparable to real acupuncture in terms of insertion depth/angle, needle manipulation, or applied electricity but without touching the targeted acupoints. In other words, the setting of the placebo acupuncture creates an exclusive window in which the acupoint is the sole explanation if the placebo and real treatments lead to distinguished outcomes, or a removal of the stimuli toward acupoints from a given procedure, the measurable clinical effects would disappear or significantly weaken.

There are some different styles of acupuncture in current clinical practice. Japanese and Taiwanese acupuncturists tend to use a relatively shallow-insertion approach, while Korean and Chinese acupuncturists prefer to use deeper needling. All the acupuncture styles' needling, however, are usually placed in the muscular layer, except for acupoints located on the top of the skull or the midline of the sternum (12, 13). The use of the above-mentioned placebo needling (Figure 1B) would likely allow acupuncture trials to be done sham-controlled, patient-blind, evaluator-blind and acupuncturist-blind as long as the needling placement sites are marked by a researcher who is the only person who knows if the sites are real or placebo, and if then the acupuncturist is told all the marked sites are known acupoints not limited to Korean, Japanese, Taiwanese, Vietnamese, French, Chinese or Western styles (14). In this scenario, the acupuncturist can be masked to group allocations which could otherwise be a source of potential bias during the research process.

Given that acupuncture is a major source of prestige in some Asian nations, some researchers conducting the acupuncture-related study might unintentionally select a suboptimal sham needling procedure (e.g., superficial needling) in an attempt to achieve more positive outcomes with traditional acupuncture (15). Both the real and placebo pills are swallowed all the way down to the stomach in the oral pharmaceutical clinical trials. But the placebo acupuncture is usually performed with a superficially inserted needle (epidermal insertion), whereas the real acupuncture needle is inserted subcutaneously (epidermal and dermal penetration, and muscular insertion); the latter, which even when missing a proposed acupoint would surely result in a comparatively stronger afferent input that could activate brainstem areas, especially the central gray matter, which are known to inhibit pain signals (16), as acupuncture is usually used for pain management.

Evidence-based acupuncture should be about the questioning of current acupuncture practice and the pursuit of the best available explanations for the mechanism of clinical acupuncture. Based on the above discussion, if future acupuncture-related experiments really want to demonstrate the specificity of acupoints, as ancient acupuncture theory claimed, giving non-acupoint and acupoint areas identical stimulus at the same-depth needling is essential.
